# Online activism and redress for institutional child abuse: function and rhetoric in survivor advocacy group tweets

**DOI:** 10.1057/s41309-022-00165-0

**Published:** 2022-04-26

**Authors:** Alasdair Henry, Katie Wright, Anthony Moran

**Affiliations:** 1 School of Global, Urban & Social Studies, RMIT University, Melbourne, Australia; 2 Department of Social Inquiry, La Trobe University, Melbourne, Australia

**Keywords:** Activism, Advocacy, Institutional child abuse, Redress, Social media, Twitter

## Abstract

In Australia, survivor advocacy groups have been closely engaged with the emergence and development of policy and redress responses to institutional child abuse. Their activities and influence in this respect have been under-researched. This study focuses on the use of Twitter, a tool increasingly employed by activist groups in their lobbying repertoires. Using content and thematic analysis, tweets of 15 non-survivor led advocacy groups, and one survivor-led organisation—*Care Leavers Australasia Network* (CLAN)—referring to ‘redress’ were analysed for rhetorical content (via Aristotle’s traditional framework of *ethos*, *pathos*, and *logos*) and communication purposes using three broad functional areas defined by Lovejoy and Saxton (2012). In keeping with Lovejoy and Saxton’s (2012) framework, the results found that for both non-survivor led advocacy groups and CLAN the primary function of their use of Twitter was to convey information to audiences. However, the integrated use of the *rhetoric* framework with the *function* framework revealed markedly different lobbying styles between the non-survivor led advocacy groups and CLAN with the latter pursuing a more confrontational and direct style of lobbying in communications. CLAN also overwhelmingly pursued emotion-focussed rhetoric in lobbying communications.

## Introduction

High-profile cases of institutional child abuse within state, church-led and community organisations have been a feature of western democracies in recent decades. In many professional and non-professional settings, physical and sexual abuse of children has been exposed as a major problem (Sullivan and Beech 1992). Global public consciousness of child sexual abuse in the Catholic Church has risen in numerous jurisdictions including Austria, Australia, Belgium, Canada, Chile, England, Germany, Ireland and Northern Ireland, and the USA (Jenkins 1998; Keenan 2011). Yet the problem of institutional child abuse extends more widely, also occurring in other religious and secular organisations (McAlinden 2013; Smith 1993), schools and residential homes (Brannan et al. 1993), and in out-of-home care (Browne and Lynch 1999).

In Australia, as elsewhere, public inquiries and other responses to institutional child abuse coincided with the formation of advocacy groups supporting victims and survivors. Since the mid-1990s, both survivor advocacy groups (formed by survivors themselves), as well as non-survivor led advocacy groups, have made submissions to public inquiries, lobbied for change, protested, participated in policy discussions, and shaped redress outcomes. Despite the prominence of these advocacy groups, there has been limited research exploring their activities and influence. Many questions are yet to be answered about activism against institutional child abuse, including the strategies, tactics, and resources employed by key advocacy groups (Wright and Henry 2019).

This article focuses on the ways that advocacy groups have utilised social media, specifically Twitter, when lobbying for redress for victims and survivors of institutional child abuse. The article develops a comparative analysis of Twitter communications about redress from one prominent survivor-led group and 15 non-survivor led organisations. Using content and thematic analysis, tweets were analysed for *function* using Lovejoy and Saxton’s (2012) three broad categories of *information*, *action* and *community,* as well as for *rhetoric* using traditional Aristotelian techniques such as *ethos*, *pathos,* and *logos* (Roberts 1984). The article then explores the interaction between *function* and *rhetoric* for all groups. Findings highlight differences between the survivor-led, and the non-survivor led advocacy groups, in their uses of rhetorical strategies and functional categories (including emotion and the ways that institutions and politicians are targeted and addressed). The fundamental role of emotion in advocacy is emphasised.

## Institutional child abuse, redress, and organised activism

Redress for survivors of institutional child abuse was the subject of the tweets analysed in this article. Australian advocacy groups had called for redress for many years before it became a key recommendation of the Royal Commission into Institutional Responses to Child Sexual Abuse (2013–2017). In July 2018, the National Redress Scheme (NRS) was established to support survivors abused in Australian institutions. Provided that an institution has ‘opted in’ to the scheme, survivors have until mid-2027 to apply to receive monetary compensation and/or psychological counselling (Department of Social Services, 2021). The NRS is not without criticism for its limitations (Wright and Swain 2021). More broadly, the scholarly literature points to multiple factors associated with the emergence of policy and redress responses to institutional child abuse. However, beyond acknowledging the centrality of survivor activism (see, e.g. Daly 2014; Golding 2010; Sköld 2013; Swain 2016) there is limited theorisation of the role of advocates and activists and how they have mobilised to effect outcomes, such as redress, for their survivor constituents (Wright and Henry 2019).

The classification of institutional child abuse advocacy groups is complex, and forms of activism vary. We categorise advocacy groups in Australia into two main types. The first is survivor-led groups—formed and coordinated by abuse survivors themselves. The second are non-survivor led groups, which typically comprise major charities (some faith-based), foundations, and community-based service providers whose organisational focus is not exclusively institutional child abuse. Many of these provide professional support services for survivors and their advocacy has extended to presenting submissions and witness statements to institutional child abuse inquiries.

While both group types have contributed to public inquires since the 1990s in Australia, it has been the coordinated activism of survivor-led groups—from diverse survivor populations such as the Stolen Generation, former Child Migrants, adult care leavers, and clerical sexual abuse survivors—that have been instrumental in instigating key inquiries about institutional child abuse. In this diverse field of actors, activism, and advocacy is not a unitary movement and there are tensions as well as alliances and partnerships in relation to the broad aim of seeking justice across this broad range of survivor populations (Wright and Henry 2019, p. 6).

Unlike their non-survivor led counterparts, survivor-led groups have typically emerged from modest beginnings and operate with limited resources, in many cases without external funding. As ‘experts of lived experience’ survivor-led groups have participated in policy making responses to institutional child abuse and government formed consultation committees about state apologies and the NRS in Australia. By contrast, many of the non-survivor led groups benefit from government funding, and revenue that enables recruitment of professional staff.

While little research exists in relation to institutional child abuse survivor-led-activism, a growing body of work exploring movements against human trafficking (e.g. Lockyer, 2020) and domestic violence (e.g. Wilson and Goodman, 2021) increasingly places value on survivor inclusion in advocacy leadership. One parallel with institutional child abuse is that the hidden nature of human trafficking results in a lack of reliable data and understanding about the phenomenon, and the inclusion of survivors in policy and program design can help address these deficiencies (Lockyer, 2020).

Self-advocacy movements are widespread and include consumer organisations responding to issues such as harmful medical procedures, mental health care, and disability discrimination. While the experiences of these groups are different, there are common characteristics (Murray, 2015 p. 164). As Allsop notes (cited in Murray 2015, p. 164) ‘People are drawn into new social movements because they feel marginalized by dominant social practices, and movements gain adherents because a positive sense of identity can develop where perceptions are shared’.

## Social media advocacy

Broader research on interest groups and social media is highly relevant for this article. On the one hand, its use as part of a group’s lobbying repertoire has tended to be seen as a ‘weapon of the weak’ (Chalmers and Shotton 2016, p. 376), an ‘outside strategy’ which, like protests and demonstrating, constitutes the means by which excluded groups attempt to make contact with decision makers (Chalmers and Shotton 2016; Edwards and Hoefer 2010; Trevor Thrall 2006, Van der Graaf et al. 2016). However, while little is known about the capability of social media to affect real political and ideological change in its intended audiences (Obar et al., 2012), growing literature highlights social media as an important advocacy tool (e.g. Bimber et al. 2012; Chalmers and Shotton 2016; Figenschou and Fredheim 2020; Guo and Saxton 2014).

Given its capacity to remove barriers between public and private spaces, social media can create new forms of participatory culture and new means for advocacy organisations to engage with their communities (Smitko 2012). In this respect they are tools for cultivating relationships, presenting new opportunities for sharing, collaborating, and mobilising collective action (Greenberg and MacAulay 2009; cited in Smitko 2012). Non-profit organisations were early adopters of social media (Greenberg and MacAulay 2009). Media studies scholars note a move to a ‘new media ecology’ framework comprising a rise in the transformation of ‘old’ technologies into a digital form enabling previously marginalised (‘non-elite’) activists increased public visibility (Chadwick 2007 cited in Pearson and Trevisan 2015, p. 925). Recent research has also considered the value of social media, particularly online communities, for abuse survivors and the potential of online spaces to function as informal sites of justice. Sexual abuse researchers have long argued that survivors have specific justice needs, and disclosing online has been described as a form of ‘cyber justice’ whereby social media is used to raise awareness about sexual violence (Powell 2015).

There has been little exploration of the use of social media by advocacy groups seeking justice for institutional child abuse. However, broader analyses and classification of the communication purposes of social media users offers useful frameworks to draw upon. Of particular note, Lovejoy and Saxton (2012) examined Twitter practices of 100 of the largest non-profit organisations in the USA. Their content analysis revealed three primary functional uses of Twitter for those organisations—*information, community,* and *action.*

The *Information* function refers to tweets ‘containing information about the organization’s activities, highlights from events, or any other news, facts, reports or information relevant to an organisation’s stakeholders’ (Lovejoy and Saxton 2012, p. 343). The *community* function identifies the use of Twitter by organisations to ‘interact, share, and converse with stakeholders in a way that ultimately facilitates the creation of an online community’ (Lovejoy and Saxton 2012, p. 343). The authors highlight two elements of the *community* function—dialogue and community building. The third function category, *action*, encourages followers to ‘“do something” for the organization—anything from donating money or buying T-shirts to attending events and engaging in advocacy campaigns’ (Lovejoy and Saxton 2012, p. 343). Where *community* is concerned, it would also make sense that advocacy groups operating in the domain of institutional child abuse would utilise social media technologies to foster a sense of connectedness between survivors, particularly where populations might be marginalised and geographically dispersed.

Relatedly, there are synergies between the *community* function highlighted by Lovejoy and Saxton (2012), and Bennet and Segerberg’s (2012) *logic of connective action* which produces a typology of *collective* and *connective* action. In its ideal form, *collective* type describes large-scale action networks that are dependent on brokering organisations to ‘carry the burden of facilitating cooperation’ (Bennet and Segerberg 2012, p. 755). *Connective* type, at the other end of the continuum, is characterised by networks that are self-organising without the steer of central or ‘lead’ organisations and where technology is critical to movement success. Examples typifying this second (connective) pattern include *Los indignados* in Spain and the *Occupy Wall Street* movement in the US, as well as the *Arab Spring* uprisings and more recently the global *#MeToo* movement. In between these two ideal types are formal organisational actors that step back from promoting political brands and strong collective agendas and identities, instead preferring to deploy social technologies to enable ‘loose public networks to form around personalised action themes’ (Bennet and Segerberg 2012, p. 757).

While the *function* of social media has been an important analytic framework, other studies have examined the ways interest groups employ *persuasion* as a strategy (e.g. Auger 2014; Higgins and Walker 2012; Pang and Law 2017). One such approach, rhetorical analysis, focuses on the overall communicative purpose of a text and its constituent parts, and the extent to which strategies of persuasion are employed (Zachary 2009). The most influential figure in the field of rhetorical analysis is Aristotle. His systematic theory of the means of persuasion associated with his major work *Rhetoric* (Roberts 1984) is foundational for many scholars working within this approach. Rhetoric, according to Aristotle, is the artful, language-based use of persuasion to effect belief (Zachary 2009, p. 71). Aristotle argues that audiences are persuaded through three means, what he calls ‘proofs’: stirring of emotions in the audience (*pathos*); force of arguments presented in the speech act (*logos*); and credibility and character of the speaker or communicator (*ethos*) (Posch 2017, p. 248).

The classical tradition of rhetorical analysis has been usefully applied to describe the interactive process employed by social media users (Pang and Law 2017). However, few studies have explored persuasion as a specific strategy. One exception, Auger (2014), employed a traditional Aristotelian framework to explore the presence of *ethos*, *pathos,* and *logos* in organisational tweets from diverse sectors, finding that each of the three ‘proofs’ were used to varying degrees to garner support and promote actions. Sixty percent of the tweets of eight charitable organisations analysed employed a rhetorical strategy and the majority of these featured a positive message frame. Similarly, in a study of two Canadian non-profit organisations (*Care2* and *United Way*), Smitko (2012) found that Twitter was used to build and strengthen relationships with donors. Rhetorical strategies were present in over 90 percent of tweets analysed, with *ethos* being the most prominent element of persuasive pattern. More recently, Pang and Law (2017) explored the use of platform features such as hashtags, URLs and visual rhetoric by activists to persuade and mobilise audiences during World Environment Day. Visual rhetoric was identified as the most prominent strategy, and the use of *pathos* and *ethos* were significantly associated with retweets.

## Method

This study focussed on tweets produced by one survivor-led advocacy group and 15 non-survivor-led groups (hereafter, NSAGs). Groups were selected based on their participation in major public inquiries into institutional child abuse held in Australia between June 1998 and December 2017. ‘Participation’ is denoted where the groups have made written and/or verbal submissions. A list of Australian inquiries was sourced from *The Age of Inquiry: A Global Mapping of Institutional Abuse Inquiries* (Wright et al. 2017). Documents produced by each inquiry were used to identify which advocacy groups had made submissions. The lead author identified a list of some 200 plus organisations contributing to 11 public inquires. This list of groups was reduced to a manageable size by including those that had contributed to more than one inquiry. This resulted in 37 groups (four survivor-led advocacy groups and 33 non-survivor advocacy groups). After determining which groups contributed to inquiries, the lead author identified which groups had active Twitter accounts and had tweeted about ‘redress’. Some groups predate Twitter and do not have active accounts. Sixteen of the 37 groups had active Twitter accounts and had tweeted about ‘redress’.

Only one survivor-led group, *Care Leavers Australasia Network* (CLAN), is included because it was the only such group with an active Twitter account who tweeted about ‘redress’. CLAN is a support and advocacy group for people who ‘have grown up in Orphanages, Children’s Homes, Missions and Foster Care in Australia and New Zealand, or whose parents or other family members had this experience’ (CLAN n.d.). CLAN has been active since 2000 and has contributed to more than six Australian child abuse inquiries. The 15 NSAGS include foundations, charities, community-based service providers, and non-profit groups whose focus, in all cases, is not exclusively institutional abuse. For example, the *CREATE Foundation* aims to ‘create a better life for children in care’, and *Relationships Australia* aim ‘to support all people in Australia to achieve positive and respectful relationships’ (Relationships Australia 2020).

Tweets including the term ‘redress’ were downloaded into a database via the Twitter application programming interface (API), using Python code specifically developed for this research. CLAN began using Twitter in 2014. The most recent 3,200 tweets for CLAN containing the term ‘redress’ were downloaded (the maximum number Twitter permits), and these all came from the period December 2018 to June 2020. Because of the Twitter restriction on the number of downloads, the period covered by CLAN’s tweets is shorter than that covered by NSAGs’ tweets. CLAN’s tweets all date from the period after the redress scheme was established (unlike NSAGs’ tweets, some of which predate the redress scheme’s establishment). For the NSAGs, the search for ‘redress’ returned a total of 157 tweets containing this term for all 15 groups and spanned the period from August 2009 to April 2020. There was notable variation in the total number of tweets from NSAGs: 10 of the 15 groups produced less than ten tweets in total containing the term ‘redress’, while the *Blue Knot Foundation*, produced 78 within the timeframe. To reduce the full dataset to a manageable size, every twenty-first tweet from the 3,200 CLAN tweets was extracted, resulting in 154 tweets for that organisation. In some cases, the entire text for tweets could not be downloaded via Twitter’s API. Incomplete tweets were not included. In total, 138 tweets produced by CLAN and 157 from the 15 NSAGs were subject to analysis. The text for all tweets, made by both group types, was imported into NVivo QSR software for coding.

The analysis addressed three key questions. First, how are survivor-led and non-survivor led survivor advocacy groups using Twitter to communicate about ‘redress’? Second, is there evidence of the employment of strategies of persuasion (rhetoric) in tweets made about redress? If so, how does this differ, if at all, between the survivor-led and non-survivor led advocacy groups? Third, how do the areas of function and rhetoric interact? A directed approach to content analysis (Hsieh and Shannon 2005, p. 1277) was employed, whereby existing theory determines the initial coding scheme, with the two key categories being *function* (Lovejoy and Saxton 2012) and *rhetoric* (Aristotle). The use of these frameworks in combination for analytic purposes was inspired by Auger’s (2014) study. Once coding was complete, codes were further grouped into key themes. Given the blend of quantitative and interpretative analysis, our methodology sits between positivist and constructivist approaches.

### Analysis of function

Tweets made by advocacy groups about ‘redress’ were coded according to their function using Lovejoy and Saxton’s (2012) framework. This approach enables analysis of the content of each tweet to determine what *communicative functions* they serve: *Action, Information, and Community*. *Action* refers to messages encouraging people ‘to do something’ for the organisation or its constituents. This can involve promotional and mobilisation uses of social media messages (Lovejoy and Saxton 2012, p. 343). *Information* covers tweets about the organisation’s activities, facts, reports, news and information relevant to stakeholders (Lovejoy and Saxton 2012, p. 343). *Community* refers to the use of Twitter to promote ‘dialogue and community building’. This includes tweets that initiate direct and interactive conversation between the organisations and their audiences, and those which serve to strengthen ties to an online community without invoking dialogue (Lovejoy and Saxton 2012, p. 343).

### Rhetorical analysis

The basic approach was to examine rhetorical performance by identifying the Aristotleian rhetorical appeals (or ‘proofs’) in tweets that relate to *ethos*, *pathos,* and *logos* (see Table 1 for summary). Persuasive appeals were coded *ethos* when they included a reference to the credibility, trustworthiness, or character of the source of the communication. Typically, the authority of the speaker or the source of the information is stressed. *Pathos* was recorded when tweets used emotion as a device to persuade audiences. Tweets were coded in this category when they contained affective appeals such as the presence of sympathy or hope and reference to underprivilege to persuade audiences. *Logos* was recorded when tweets included data or evidence in support of their message. Given that *Logos* refers to the ‘clarity and integrity of the argument’ (Holt and Macpherson 2010 cited in Pang and Law 2017, p. 57), tweets that presented components of reasoning and logic within argumentation styles were also included.

**Table 1:** Elements of rhetorical appeals. Adapted from Pang and Law, (2017, p. 57)

Appeal	Examples of persuasive techniques
Ethos	Perceived character; reference to authority figure or expert
Pathos	Sympathy; Hope; Under-privilege
Logos	Argumentation; Evidence; Case study; Data

## Results

### Function of twitter use

#### Information

As with previous research utilising Lovejoy and Saxton’s (2012) framework, we found that for all groups, the primary function of Twitter was the dissemination of information. For CLAN, 72 of 138 tweets (52%) were categorised as information, and for the NSAGs 89 of 157 tweets (59%) were coded in this category (Table 2).

**Table 2:** Function of Twitter Use

Function	Number		Percentage of tweets (made by groups)	
	CLAN	NSAGs	CLAN	NSAGs
Information	72	89	52%	59%
Action	53	62	38%	39%
Community	13	6	10%	2%
**Total**	**138**	**157**	**100%**	**100%**

Bold is used here to highlight totals

For the NSAGs, the information function of Twitter was used primarily to convey information about redress scheme developments. Within this category the most frequently used (at 31%) information tweets communicated news updates about policy and redress scheme developments such as announcements about legislation enacting the implementation of the NRS and news of institutions signing on to the scheme. The second most frequently used (at 11%) theme for the NSAGs within *information* tweets offered procedural advice to survivors on applying to the NRS. Smaller frequencies were recorded across a range of themes including information about counselling services and organisation-specific activities (e.g. advertising jobs within the organisation, notifications of social events, and linking audiences to other social media posts). From the tweets made by the non-survivor led groups it is possible to infer that there is a wide intended audience. These include other survivor advocacy organisations, media, policy makers and members of parliament.

Many of these themes within the *information* category were also present for CLAN, including information specifically targeted at service users about redress process developments which constituted the most frequently used theme within *information* at 22%. These types of tweets are frequent probably because CLAN’s Twitter audience includes many survivors. As with the NSAGs, there were also many (13%) tweets reporting the activities of ‘care’ institutions in respect of their participation (or not) in the NRS. Other *information* focussed tweets from CLAN highlighted relevant media releases (e.g. news articles about redress), as well as information about CLAN’s social events.

#### Action

For both CLAN and the NSAGs, the second most frequently coded Twitter function was *action.* Both CLAN and NSAGS recorded equivalent frequencies of action focussed tweets at 38% and 39%, respectively. For NSAGs, the common themes within tweets stressed ‘actioning a redress system immediately’. Many of these tweets were directed at policy makers, although the use of the ‘@’ symbol to direct tweets at political figures featured in only three *action* tweets from NSAGs. The earliest recorded reference to ‘redress’ made by one of the NSAGs was in August 2009. However, calls for redress intensified in late 2016 in the lead up to federal government announcing the scheduled July 2018 implementation of the NRS. Multiple tweets called for bipartisanship and government unification on redress scheme planning and implementation:

Federal Govt should lead bi-partisan support to develop, implement and fund any redress scheme (Australian Association of Social Workers, Aug 20, 2015).

The Turnbull government must not back away from a national redress scheme. (Blue Knot, Jan 14, 2016).

In the period post-June 2018, after the establishment of the NRS, the second most common theme within action tweets for NSAGs was to urge state governments and former care institutions to join the scheme; this was found in 15 *action* tweets about redress. In addition, tweets frequently stressed the need to create an equitable and inclusive redress system, one which would not exclude claimants and survivors with criminal records.

For CLAN, the vast majority of *action* tweets addressed government members directly via the use of the ‘@’ symbol in tweets. Indeed, the top five most frequently used ‘@’ addresses by CLAN were all linked to politicians. The federal government, which created the scheme, was overwhelmingly the target of *action* category CLAN tweets. The most common theme in these demands for action related to CLAN urging government and individual elected officials to financially penalise institutions reluctant to sign up to the NRS. Institutions not ‘opting in’ were frequently referred to as ‘redress laggers’ in tweets and CLAN argued that they should be ‘named and shamed’ by the government:

If the Churches Charities Redress laggers won’t join #Redress. Governments must introduce a #ChildAbuseTAX @billshortenmp @ScottMorrisonMP @PaulFletcherMP @LindaBurneyMP.

(CLAN, March 25, 2019).

@Anne_Ruston Why wait…?Name &; [sic] Shame #Redress Laggers NOW 9 orgs said they won’t join Strip charity tax from abusers No more carrots. Get the sticks canes feather dusters switches straps &; [sic] bats they used on us children Fancy giving abusers options.

(CLAN, Feb 27, 2020).

The second major focus of calls for action, directed at the government from CLAN, related to speeding up redress processes for survivors, increasing payments, and calls to cease the practice of ‘indexing’ (ranking payments according to nature and extent of abuse experienced):

@ScottMorrisonMP @billshortenmp.Just spoke to CLAN’s oldest member 97ysold Katie still waiting for #Redress Why the wait, #Nuns have joined ? Is it snail pace cos of #Elections2019 @PaulFletcherMP @LindaBurneyMP @SharonClaydon @HumanHeadline @SenClaireMoore @jennymcallister.

(CLAN, Feb 25, 2019).

Another important target for *action* related tweets made by CLAN were former care institutions. Tweets coded in this category demanded that institutions join the NRS. The tone of tweets directed at institutions was frequently confrontational:

@RedCrossAU When are joining Redress ? CareLeavers abused in your #children’sHome they are waiting for you to find your moral compass.

(CLAN, March 29, 2019).

#### Community

Tweets were coded as *community* when there was evidence of the use of Twitter to promote dialogue and community building. Of all three function categories, for both CLAN and NSAGs, this appeared least frequently, however the incidence of community focussed dialogue was considerably higher for CLAN (10% of tweets) than for NSAGs (at 2%). *Community* focused tweets for CLAN typically thanked CLAN members for their activism and advocacy, rallying and encouraging members to engage in activist events such as protests:

Thankyou #Clannies for retweeting CLANs tweets. Your tweets mean a lot &; [sic] it keeps pressure on the pollies &; [sic] Govts to do the right thing by us Hurry up Believe #CareLeavers Pay #Redress delays B4gets CLs Clannies please keep retweeting we appreciate your support.

(CLAN, April 5, 2020).

### Rhetoric: strategies of persuasion

Coding identified the presence of rhetorical ‘proofs’ in 97% of tweets made by CLAN and 63% for the NSAGs. For the NSAGs the remaining 37% of tweets were made up exclusively of *information* communications. Among these, the most common themes were news updates about redress scheme developments, information directed at redress scheme users in relation to application processes and announcements about counselling services offered by NSAG organisations for abuse survivors. The presence of multiple rhetorical appeals (more than one per tweet) was not found in any tweets (Table 3).

**Table 3:** Rhetorical Appeal

Rhetorical Strategy	Number		Percentage of all tweets	
	CLAN	NSAGs	CLAN	NSAGs
Ethos	6	26	4%	17%
Pathos	84	19	61%	12%
Logos	44	55	32%	35%
**Total**	**134**	**100**	**97%**	**63%**

Bold is used here to highlight totals

#### Pathos

There was a dominant presence of *pathos* in tweets made by CLAN, which was detected in 61% of all tweets made by the group about redress. Tweets were coded in this category when they contained emotionally affective appeals and where there were a range of emotions—overwhelmingly negative—articulated by CLAN in their persuasion repertoires. The most common theme in *pathos* was about care leaver survivors. These tweets often included reflections about experiences of abuse while in ‘care’ and about how redress delays were another form of systemic institutional abuse. They also highlighted how the redress system was creating a sense of abandonment among care leaver claimants, particularly the elderly, who were left waiting for payouts:

Child sexual abuse #CareLeavers &; [sic] others waiting 'too long' for compensation under Australia's redress scheme…Elderly are at great risk of dying #Coronavirus.

(CLAN, Apr 30, 2020).

Positive emotions within *pathos* persuasion strategies included motivating and rallying messages urging survivors to ‘keep up the fight’, as well as expressing gratitude towards survivors who had been campaigning for redress on behalf of adult care leavers. Government and ‘care’ institutions were frequently the focus of tweets containing *pathos* rhetoric and which typically articulated angry and frustrated sentiments towards both parties. Retribution was a common theme in tweets directed at institutions promising that survivors would eventually get their ‘just deserts’ as well as stressing that care providers had a moral obligation to participate in the NRS. Anger, frustration and disgust were evident in criticism of the redress scheme itself and its perceived failure to satisfactorily (and speedily) compensate claimants:

47 days til Minister @Anne_Ruston names &; [sic] shames the #Redress Laggers on 30th June 2020. Polish Order of #Nuns ran 2 Children’sHomes in Australia. #Resurrection House #Essendon Vic. St Stanislaus #RoyalPark #Adelaide. Show your moral compass Sisters.

(CLAN, May 13,2020).

For the NSAGs, *pathos* was recorded in 12% of all tweets, constituting its least frequently utilised rhetorical appeal. Like CLAN a major theme in this category for the NSAGs was to underscore how delays and failures with redress were resulting in a sense of that survivors were being abandoned. However, unlike CLAN tweets about delay and abandonment issues, blame for this was not directed specifically at government figures or institutions:

Survivors can't wait any longer. Time for fair equitable redress overdue.# redress; #national redress #redressscheme.

(Blue Knot, Feb 18, 2018).

NSAGs’ *pathos* tweets also differed from CLAN’s because they were more likely to utilise positive emotions in their persuasive strategies. Numerous tweets directed emotions of gratitude towards governments and institutions where progress had been made:

Congratulations to the Qld Gvt for passing legislation today to allow redress to proceed for thousands of Queensland child sex abuse victims. "Redress is about healing, justice and recognising past wrongs”.

(Micah Projects, Sept 19, 2018).

#### Logos

For NSAGs by far the most frequently coded rhetorical proof was *logos*, found in 35% of all tweets. Tweets categorised as *logos* demonstrate a particular focus on the clarity and integrity of the argument in support of the position the tweet is contending. While NSAGs made a range of diverse arguments about redress, four clear themes emerged. The most frequently *logos* coded tweets were arguments insisting that the proposed redress scheme should ensure equal access for all survivors, regardless of the type of abuse experienced, or whether survivor claimants have criminal records:

Consistency and fairness needed in national redress scheme to better support survivors @CARoyalComm.

(Braveheart, March 31, 2015).

The second most frequently coded theme in *logos* for the NSAGs was the argument that institutions sign up to the redress scheme as a matter of moral responsibility:

There is no excuse for institutions to not join the redress scheme. Time to stand up and be counted. Survivors of child sexual abuse waiting too long for redress scheme…

(Blue Knot, April 29, 2020).

Four tweets from NSAGs made arguments calling for urgent federal government action to implement a national redress system, and four others urged bipartisanship and the ‘removal of party politics’ where decisions about redress system implementation were concerned:

Redress scheme for #childsexualabuse victims who are part of the @CARoyalComm should be 'above party politics’.

(Child Wise, May 10, 2016).

For CLAN *logos* was recorded as the second most frequently used rhetorical ‘proof’ found in 32% of all tweets made about redress. In some cases, CLAN employed data or evidence to support their arguments, but more often (like the NSAGs) where *logos* was recorded their approach revealed reasoning and logic within argumentation style as a form of persuasion.

Arguments coded in *logos* covered a range of issues concerning redress scheme inadequacies and perceived injustices. These included that redress should focus on a range of abuses (not exclusively child sexual abuse), that survivors living outside of Australia were being unfairly rendered ineligible, that redress payments be expediated, and that the monetary amounts were insufficient. The most frequently recurring *logos* argument by CLAN was insistence that ‘redress ‘laggers’ be penalised for failing to commit to the NRS, for example, through removal of their charitable status:

#DSS states it would be counterproductive to name #Redress Laggers Yet the Minister @Anne_Ruston said she will name the organisations. There are 295orgs have NOT joined NO more funding laggers remove #charitytax.

(CLAN, Mar 19, 2020).

#### Ethos

The least frequently coded rhetorical appeal for CLAN was *ethos* (found in 4% of all tweets), that is, a rhetorical proof that included a reference to trustworthiness or credibility. CLAN tweets coded in this category made reference to academic publications, media, senate documents, and abuse survivors themselves in the articulation of persuasive tweets about a range of issues pertaining to redress:

Dear Minister &; [sic] Premier. This paper on Orphanage #Education presented 1994 Conference of Australian Ass for Research in Education by David Maunders will help u to understand the #Redress application disadvantages #CareLeavers.

(CLAN, Jun11, 2019).

NSAGs were much more likely than CLAN to rely on *ethos* as an appeals category in their rhetoric (found in 17% of all tweets). The most frequently coded strategy in *ethos* for NSAGs was to rely on politicians as sources of trustworthiness when making announcements about redress developments. The notable theme here is that when politicians were cited as sources of credibility, trustworthiness was not scrutinised as it had been vociferously in tweets made by CLAN:

Australian PM @TurnbullMalcolm pledges redress scheme proposed by @CARoyalComm for victims of #childsexual abuse. PM's pledge to victims of abuse Malcolm Turnbull has promised a group of child abuse victims that I will not let you down.

(Child Wise, Sept 20, 2016).

The second most frequently recorded theme in *ethos* for NSAGs was a reliance on academics in persuasive tweets about redress:

Any redress scheme should be done on a national basis’ @qutlaw Professor Ben Mathews talks to @RadioNational #RoyalCommission’

(Bravehearts, May 10, 2016).

### Interaction between function and rhetoric

The article now turns to examining the relationship between the use of rhetoric and the three broad areas of Twitter function: *action*, *information,* and *community.* Tables 4 and 5 illustrate distribution and variation of rhetorical proofs within the three functional areas. Overall, for CLAN *pathos* in the *information* category followed by *pathos* in the *action* category were the most frequently used rhetoric and function combinations. The least frequently coded combination was *logos* and *ethos* in the community category where no rhetorical appeals were recorded. For the NSAGs, the most frequently coded combination category (n = 46) was *action* and *logos* followed by *information* and *ethos* (n = 16)*.* No rhetorical appeals were present in the *community* category for the NSAGs.

**Table 4:** Interaction between Function and Rhetorical Appeals – CLAN Value = Frequency (and percentage of total CLAN tweets)

	Action	Information	Community	*Total*
Logos	21 (16%)	23 (17%)	0 (0%)	*44 (32%)*
Ethos	2 (1%)	4 (3%)	0 (0%)	*6 (4%)*
Pathos	25 (18%)	54 (39%)	5 (4%)	*84 (61%)*
**Total**	**48 (35%)**	**81 (59%)**	**5 (4%)**	**N = 134 (97%)**

Bold, italics are used here to highlight totals

**Table 5:** Interaction between Function and Rhetorical Appeals – NSAGs Value = Frequency (and percentage of total NSAG tweets)

	Action	Information	Community	*Total)*
Logos	46 (29%)	9 (6%)	0 (0%)	*55 (35%)*
Ethos	10 (6%)	16 (10%)	0 (0%)	*26 (17%)*
Pathos	5 (3%)	14 (9%)	0 (0%)	*19 (12%)*
**Total**	**61 (39%)**	**39 (25%)**	**0 (0%)**	**N = 100 (64%)**

Bold, italics are used here to highlight totals

Perhaps the most notable finding within the interaction data relates to the differences between NSAGs’ and CLAN’s use of rhetoric within the *action* category. As highlighted above, both CLAN and NSAGs used Twitter for *action* at similar frequencies (i.e. 38% and 39% of all their tweets, respectively). As per Table 5, NSAGs were much more likely to employ *logos* rhetoric to persuade audiences about what ‘should be done’ (actioned) in relation to redress at a frequency of 29% of all tweets. This was nearly twice that of CLAN, who recorded the same combination in 15% of tweets. However, CLAN were six times more likely than NSAGs to employ *pathos* as a strategy to persuade audiences about what should be actioned in relation to redress (18% versus 3%, respectively), suggesting markedly different persuasive strategies where *action* tweets were concerned.

Further, the element of *emotion* was also highly evident in the *information* category itself, whereby we identified a distinct theme—*expression of emotion*. This code was reserved for any tweets that were employed solely for the purpose of conveying emotional states. Primarily these tweets were used for the purpose of ‘venting’ and expressing frustration, anger, sadness, or sentiments of shame. These types of tweets made up 28% of CLAN’s *information* tweets and could arguably constitute an autonomous category of Twitter *function*.

## Discussion

As with Lovejoy and Saxton’s (2012) study, we found that social media was used across the groups primarily for the purpose of conveying information to audiences. However, it is clear that Twitter was also used for the purposes of lobbying, for example, through appeals to audiences about ‘what should be done’ with regard to redress. Both group types within the *action* category sought similar outcomes—an equitable and speedy delivery of payments, universal redress scheme ‘opt in’ from institutions and state governments, and bipartisan leadership around a national scheme were all shared themes. There was also a strong presence of rhetoric across all groups. For the NSAGs rhetorical appeals were identified in 63% of their tweets, which aligns with Auger’s (2014) finding (at 60%) for the selected non-profit groups in that study, which used the same analytical framework. For CLAN, there was a much stronger presence of rhetoric—found in 97% of all tweets.

The critical findings in this study relate to markedly different styles of lobbying identified within tweets made by CLAN and the NSAGs. Firstly, CLAN employed a more direct lobbying strategy in that they made specific demands to clearly identifiable audiences. The use of the ‘@’ symbol was prominent in tweets made by CLAN and the top five most frequently used ‘@’ handles in all their tweets linked tweets directly to MPs. By contrast, only on three occasions were requests made by the NSAGs for ‘something to be done’ directed at specific individuals. Where individual MPs were cited, it was to offer praise where their redress related activities were perceived in a positive light. In short, the target audience for NSAG tweets where lobbying efforts were employed was more ambiguous than CLAN’s tweets, with CLAN preferring to directly lobby individuals (particularly elected officials) and the NSAGs being much less direct. As an organisation, CLAN has enjoyed considerable success in working directly with politicians across major political parties in Australia, with a number of these being patrons of the group (Murray 2015). This may explain CLAN’s willingness to attempt to engage so directly with MPs in their lobbying about redress.

A common theme in CLAN’s tweets was to negatively critique the perceived inactivity of authorities. Rarely (although not absent entirely) did CLAN tweet praising institutions or individuals in authority for acting in ways perceived to be positive for survivors and the redress process. The most aggressive and critical-in-tone remarks were reserved for the former ‘care’ institutions themselves, where frequently a strong retributive tone was employed in CLAN’s demands for ‘redress laggers’ to be financially penalised and/or publicly shamed.

CLAN was also overwhelmingly (five times) more likely to employ emotional (*pathos*) rhetoric in the delivery of persuasive tweets compared to NSAGs. The dominant presence of less positive emotions such as anger and frustration from CLAN were anticipated given that the group speaks directly on behalf of its survivor constituents and are redress system users themselves. By contrast, the NSAGs were much more likely to use *ethos*, i.e. the character and credibility of the sources of information they are using to argue about ‘what should be done’.

It is unsurprising that CLAN sought to employ emotion as a primary resource in its lobbying repertoire about redress. This deployment of *pathos,* in interaction with *information* and *action* is a strategy of self-advocacy and these are emotions that CLAN, as survivors themselves, can authentically articulate in pursuit of their own needs and interests about redress. Parallels can be drawn here in relation to the self-advocacy of survivors in anti-human tracking and domestic abuse social movements who have also long sought for the inclusion of their voices, experiences and perspectives to inform policy development and service provision. The literature about anti-trafficking movements highlights the importance of survivor perspectives in more accurately and effectively guiding policy and program decisions (Lockyer, 2020). A growing body of research also suggests that survivor self-advocates in the movement against domestic violence derive growth and meaning from their advocacy which presents the opportunity to validate survivorship (Wilson and Goodman, 2021). It is also possible that such differences in the rhetoric employed by the groups could be a function of the level of professionalism within the organisation. For example, it may be that groups with non-survivor staff responsible for social media accounts are less likely to use *pathos* and more likely to rely on *ethos*, or that the emotions displayed are different if the person running the social media account for a group is an employee versus a volunteer.

While this study highlights clear differences between CLAN and non-survivor led groups in this domain, it is also important to remember that there are differences both between CLAN and NSAGs as well as *among* the NSAGs where strategies and organisational objectives are concerned. The key finding here is that CLAN and NSAGs rely on different resources in their online lobbying strategies and efforts to persuade audiences. The literature addressing the role of emotions in social movements tells us that perceived injustices can evoke emotions which may instrumentally serve to recruit and mobilise sympathetic bystanders (Van Ness and Summers-Effler 2019). When activists have an identifiable target for blame, identification with one’s in-group and feelings of solidarity and camaraderie can manifest (Van Ness and Summers-Effler 2019 p. 414). In this respect, the range of perceived violations of survivors’ expectations in relation to redress, articulated by CLAN in their tweets, is arguably intended to invoke sympathy for the cause. After all, the primary aim of many of these targeted tweets is to garner support from specific MPs so that they may exert pressure (from the inside) on policy makers, as well as on the outside at former ‘care’ institutions and churches.

Further research might explore the impact of emotions on target audiences employed in social media spaces by advocacy groups in the institutional child abuse domain and beyond. Turner and Stets (2005) make a critical distinction between primary and secondary emotions, with the former being fear, anger, happiness and sadness. Secondary emotions relate to more socially constructed variants such as shame, pride, disappointment, alienation, anxiety, and indignation, for example. Many of these emotions were employed in rhetoric used by CLAN, and by the NSAGs also, albeit to a less extent. Turner and Stets (2005) argue that humans are responsive to these emotions (particularly the primary ones) in face-to-face settings and involvement during protests (Ness and Summers-Effler 2019, p. 414). Future research might explore the extent to which lobbying targets are responsive to these different emotional states expressed through social media platforms.

Scholars examining social movements also tend to overlook the role of negative emotions such as anger and retribution and therefore fail to highlight the binding significance these emotions can perform for group members (Jasper 2014). It may be that many of the negative emotions expressed by the groups work in constructive ways, and some have argued that group-based anger is an important motivator of protest mobilisation for disadvantaged groups: ‘People who perceive the in-group as strong are more likely to experience anger and desire to take action; people who perceive the in-group as weak are more likely to feel fearful and to move away from the out-group’ (van Stekelenburg and Klandermans 2013, p. 893). Future research might therefore also explore the potential for these types of emotions to constructively forge collective identities within abuse survivor advocacy groups and the role social media platforms might play in this.

## Conclusion

The function framework inspired by Lovejoy and Saxton (2012) and employed in this study revealed that for both CLAN and NSAGs the primary function for Twitter was to convey information to audiences. Similar frequencies within the *action* category were also recorded, highlighting that both group types, when talking about redress, were also using Twitter for lobbying purposes. The integrated use of the *rhetoric* framework with the *function* framework revealed markedly different lobbying styles between CLAN and the NSAGs. CLAN pursued a more aggressive, confrontational, and direct style of lobbying compared with their non-survivor led counterparts. This is evident in several ways. Typically tweets made by CLAN explicitly identified target audiences (primary MPs and ‘care’ institutions) in its action/lobbying communications. In addition, critical and retributive tones were frequently evident in CLAN tweets, particularly when rhetoric was directed at institutions where they were perceived as ‘redress laggers’ for example, and at government when seen to be failing to act in the best interests of survivors and redress claimants.

CLAN also overwhelmingly pursued emotion-focussed rhetoric in lobbying communications. Where Twitter communications were *information*, *pathos* also dominated this category, and it was clear that an important function of Twitter for CLAN was to vent frustrations about difficulties endured by those navigating the redress system and seeking justice. The NSAGs by contrast (while just as likely to be using Twitter for lobbying focussed communications) were much more ambiguous in respect of their intended target audiences and were also six times less likely to rely on emotion focussed rhetoric in their lobbying strategies. They were also less likely to be critical of specific audiences, preferring instead to be praising of government and institutions when they were perceived to be acting in ways favourable to abuse survivors and redress claimants.

Bennett and Segerberg’s (2012) *logic of connective action* distinguishes between self-organising and technology-led online activism. Advocacy about redress for institutional child abuse is *not* self-organising and activism from established interest groups has been critical in the emergence of redress responses to institutional abuse. Our study highlights the ways that interest groups (both survivor and non-survivor led), broker networks or use social technologies to form public networks. CLAN’s use of Twitter in relation to redress is characterised by strong organisational coordination in attempting to build interpersonal connections (particularly via ‘@’ targeted tweets) to directly lobby politicians. Rather than seeking to lobby officials directly, the NSAGs by contrast sought to form loose social networks encouraging publics to engage with one another in action against redress.

Social media strategies have been, in some quarters, regarded as a ‘weapon of the weak’ (Chalmers and Shotton 2016), but it is also clear that they have played a critical role in the emergence of major social movements globally. This study shows how the prominent Australian advocacy group, CLAN, uses Twitter as a form of activism, underscoring the different online lobbying styles used by a survivor-led group and other advocacy organisations. Further research exploring the impact of such approaches on their target audiences in the institutional child abuse domain would be highly illuminating particularly with regard to the use of emotion as a persuasive strategy. The sociology of social movements underscores the importance of emotion. Emotions permeate political action, and this study reveals that advocacy groups are using social media to mobilise emotion to garner support and lobby politicians about redress for institutional abuse survivors.

## Survivor advocacy groups included in study

### Survivor-led Group

- Care Leavers Australasia Network (CLAN)

### Non-Survivor-led Groups (NSAGs)

- Australian Association of Social Workers
- Anglicare (Australian Capital Territory)
- Anglicare (South Australia)
- Blue Knot Foundation
- Bravehearts
- CASA Forum
- Child Wise
- CREATE Foundation
- Healing Foundation
- Interrelate
- Micah Projects
- People With Disabilities Australia
- Relationships Australia
- Survivors and Mates Support Network
- Truth Justice Healing Council

### Term Frequencies—NSAGs

**Top hashtags (of 157 sample tweets containing term ‘redress’)**

1. #Redress (25) -
2. #royalcommission (11)
3. #protectkids (5)
4. #CAroyalcommission (4)
5. #survivors (4)
6. #childabuse (3)
7. #nationalredress (3)
8. #respect (3)
9. #abuse (1)
10. #aupol (1)

**Top ten ‘@’ (targeted address of tweet containing term ‘redress’)—Frequency and Target Description**

1. @blueknotorg (80)—Survivor Advocacy Group
2. @pwdaustralia (20)—Survivor Advocacy Group
3. @micahprojects (17)—Survivor Advocacy Group
4. @caroyalcomm (14) – Royal Commission into Institutional Responses to Child Sexual Abuse
5. @tjhcouncil (14)—Survivor Advocacy Group
6. @braveheartsInc (10)—Survivor Advocacy Group
7. @aasw (9)—Survivor Advocacy Group
8. @annastaciamp (9) – Member of Parliament
9. @createfnd (7)—Survivor Advocacy Group
10. @ascaorg (6)—Survivor Advocacy Group

### Term Frequencies—CLAN

**Top hashtags (of 138 sample tweets containing term ‘redress’)**

1. #Redress (138)
2. #Careleavers (33)
3. #auspol (23)
4. #nuns (13)
5. #springst (10)
6. #royalcommission (6)
7. #children’shomes (5)t
8. #Salvationarmy (5)
9. #statewards (5)
10. #sydney (5)

**Top ten ‘@’ (targeted address of tweet containing term ‘redress’):**

1. @Anne_Ruston (47)—Member of Parliament
2. @lindaburneymp (26)—Member of Parliament
3. @scottmorrisonmp (24)—Member of Parliament
4. @paulfletchermp (21)—Member of Parliament
5. @jillhennessymp (21)—Member of Parliament
6. @abcnews (11)- Media outlet
7. @deansmithwa (10) – Member of Parliament
8. @clan (10) – Survivor advocacy group
9. @humanheadline (10) – Media Personality
10. @sharonclaydon (10) – Member of Parliament
